# The mode and tempo of genome size evolution in the subgenus *Sophophora*

**DOI:** 10.1371/journal.pone.0173505

**Published:** 2017-03-07

**Authors:** Carl E. Hjelmen, J. Spencer Johnston

**Affiliations:** Department of Entomology, Texas A&M University, College Station, Texas, United States of America; Fralin Life Science Institute, Virginia Tech, UNITED STATES

## Abstract

Genome size varies widely across organisms, with no apparent tie to organismal complexity. While genome size is inherited, there is no established evolutionary model for this trait. Hypotheses have been postulated for the observed variation in genome sizes across species, most notably the effective population size hypothesis, the mutational equilibrium hypothesis, and the adaptive hypothesis. While much data has been collected on genome size, the above hypotheses have largely ignored impacts from phylogenetic relationships. In order to test these competing hypotheses, genome sizes of 87 *Sophophora* species were analyzed in a comparative phylogenetic approach using Pagel’s parameters of evolution, Blomberg’s K, Abouheif’s C_mean_ and Moran’s I. In addition to testing the mode and rate of genome size evolution in *Sophophora* species, the effect of number of taxa on detection of phylogenetic signal was analyzed for each of these comparative phylogenetic methods. *Sophophora* genome size was found to be dependent on the phylogeny, indicating that evolutionary time was important for predicting the variation among species. Genome size was found to evolve gradually on branches of the tree, with a rapid burst of change early in the phylogeny. These results suggest that *Sophophora* genome size has experienced gradual changes, which support the largely theoretical mutational equilibrium hypothesis. While some methods (Abouheif’s C_mean_ and Moran’s I) were found to be affected by increasing taxa numbers, more commonly used methods (λ and Blomberg’s K) were found to have increasing reliability with increasing taxa number, with significantly more support with fifteen or more taxa. Our results suggest that these comparative phylogenetic methods, with adequate taxon sampling, can be a powerful way to uncover the enigma that is genome size variation through incorporation of phylogenetic relationships.

## Introduction

When considering trait evolution, sequence of the genotype is traditionally inspected for evidence of selection or drift, through methods such as DN/DS ratios. These tests, however, are not easily applied to genome size. Genome size, like gene expression, is an intermediate phenotype; while the trait is directly influenced by the sequences in the genome, there is not a specific sequence tied to it, and it must therefore be analyzed in a phenotypic fashion. Genome size has been found to vary up to 200,000-fold in eukaryotes [1] and up to 7,000-fold in animals [2], and seems to bear no correlation with organismal complexity among eukaryotic taxa. The wide variation in genome size is not generally attributed to coding DNA sequences, but rather to repetitive and nongenic DNA [1, 3, 4]. For these reasons, we are analyzing the variation in DNA content in a comparative phylogenetic context with the goal of establishing an evolutionary model for genome size from proposed hypotheses.

Among the many questions that remain to be answered in the field of genome size evolution is what drives or constrains genome size [5]. One of the most fundamental questions asks, “Is the accumulation of nongenic DNA adaptive or just tolerated by selection?” and, “If the accumulation is adaptive, what benefits does it have? What mechanisms underlie genome size change?” Of the many hypotheses for genome size evolution (reviewed in [1]), we focus on the effective population size hypothesis [6, 7], mutational equilibrium hypothesis [8–10], and the adaptive hypothesis of genome size evolution [11–13].

Lynch and Conery propose that changes in genome size occur primarily during speciation events due to coincident small species level effective population sizes [6, 7]. They argue that selection is ineffective at lower effective population sizes, and therefore potentially maladaptive changes in genome size may accumulate and persist in the population (e.g. increases in genome size from transposable elements). In contrast, species with large effective population sizes will be less likely to tolerate large changes in genome size, due to more effective selection.

The mutational equilibrium hypothesis proposes that genome size change is gradual and is due to an imbalance in indels (insertions and deletions) that through time, eventually achieve a mutational equilibrium [8–10]. Some genomes are favored to move towards smaller sizes because they tend to have higher deletion rates compared to insertion rates [14]. The change, whether downwards or upwards, can be considered gradual, yet proportional; larger genomes can handle larger insertions and deletions than smaller genomes. The above hypotheses suggest different rates and modes of phylogenetic change. The effective size hypothesis would produce change early in the speciation process, when species effective size is small. In contrast, an insertion deletion balance could increase or decrease genome size, yet the change would accumulate gradually over phylogenetic time. While proposed to explain the variation in genome size, some consider this hypothesis to be largely theoretical and yet to be supported by a large dataset [15, 16]. Recently, a related accordion model, which proposes that increases in DNA content by transposable elements is balanced by large segment deletions, was supported by data from 10 species of mammals and 24 species of birds [17]. The model differs from the mutational equilibrium model only in that the change involve larger segments and have the potential for faster rates of evolution.

A third hypothesis, that of adaptive genome size evolution, was summarized by Powell [13] and is reviewed in Gregory [18] and Gregory and Johnston [12]. The adaptive hypothesis proposes that genome size should track the environment; environmental change results in genome size change. Because species evolve to utilize habitats uniquely, we would expect adaptation to uncouple genome size and the phylogeny. While there could possibly be some phylogenetic signal throughout time in this hypothesis, when organisms shift to new ecological environments, punctuated shifts in signal are expected. As such, adaptive changes are unlikely to present clear phylogenetic patterns. It is important to note, however, that if these shifts in habitat that drive genome size change are shared by members of a clade, the relationship between adaptation and signal may be difficult to untangle.

Even though much data has been accumulated for genome sizes (5,635 species to date according to genomesize.com [19], the ever present genome size variation has been largely ignored from a phylogenetic standpoint. The importance of this issue is highlighted in a phylogenetic analysis of the data presented as proof of the relationship of species effective size and genome size change [6, 20]. The proposed significant relationship between effective population size and genome size is lost when accounting for the phylogeny, leaving Lynch’s effective population size hypothesis for genome size evolution conjectural. In general, the lack of phylogenetic consideration has resulted in a lack of knowledge about how changes in genome size have occurred throughout evolutionary history, whether random or adaptive and selected.

In an effort to address the lack of consideration of phylogenetic relationships among species when analyzing genome size variation, we produced a phylogeny of Drosophilidae, with a focus on *Sophophora*, using aligned sequence data for 87 species. The resulting tree and associated branch lengths was used to generate Pagel’s parameters of evolution, Blomberg’s K, Moran’s I, and Abouheif’s C_mean_, for genome size evolution in *Sophophora* [21–24]. If Pagel’s λ and Blomberg’s K are approaching one, the presence of phylogenetic signal would suggest that genome size is not evolving according to the adaptive hypothesis. If there is signal, and Pagel’s κ and δ values are approaching 1, this would suggest gradual change of genome size throughout time, supporting the mutational equilibrium hypotheses. If there is signal, with δ and κ values below one, this would suggest early change in branches and the tree, supporting the low effective population hypothesis.

The complete analysis utilized a relatively large number of species (87) and a 3X range of genome size values to generate estimates. To determine the reliability of these phylogenetic analyses with different numbers of taxa, we generated the same parameter estimates with reduced taxa numbers and reduced ranges of genome size. Several, but not all of the parameters are sensitive to taxon number; genome size range had little effect on the results.

## Materials and methods

### Genome size database

Genome sizes for species were obtained from published datasets [12], with additional data from the laboratory database of J. Spencer Johnston. Genome sizes were estimated using the flow cytometric method [25] for species obtained from the UC San Diego Species Stock Center (http://stockcenter.ucsd.edu) (Table 1).

**Table 1:** Genome size estimates for 87 species of Drosophilidae.

Stock Number	Species	Genome Size	Stock Number	Species	Genome Size
20010–2010.00	*C*. *amoena*	395.2	14028–0651.00	*D*. *triauraria*	245.5
20000–2631.01	*C*. *procnemis*	280.1	-	*D*. *quadraria*	252.1
13000–0081.00	*D*. *busckii*	139.9	14028–0661.03	*D*. *rufa*	255.4
-	*D*. *mauritiana*	157.9	14028–0591.00	*D*. *mayri*	257.5
14021–0224.01	*D*. *erecta*	158.9	14029–0011.00	*D*. *fuyamai*	264.1
14021–0251.195	*D*. *simulans*	159.6	14028–0481.00	*D*. *baimaii*	278.4
14028–0541.00	*D*. *kanapiae*	165.5	-	*D*. *orena*	280.7
-	*D*. *teissieri*	166.3	-	*D*. *lucipennis*	291
14021–0248.25	*D*. *sechellia*	166.7	14028–0731.00	*D*. *pectinifera*	297.4
-	*D*. *varians*	166.7	-	*D*. *suzukii*	342.8
14021–0261.01	*D*. *yakuba*	170.7	-	*D*. *pseudoobscura*	167.7
-	*D*. *santomea*	171.5	-	*D*. *miranda*	175.6
4021–0231.36	*D*. *melanogaster*	174.5	-	*D*. *tolteca*	179
14025–0441.05	*D*. *ficusphila*	190.8	-	*D*. *ambigua*	186.8
-	*D*. *birchii*	191.2	14011–0111.49	*D*. *persimilis*	197.1
14027–0461.03	*D*. *elegans*	192.2	-	*D*. *azteca*	199.5
-	*D*. *pallidosa*	194.1	-	*D*. *affinis*	200.5
14024–0361.00	*D*. *atripex*	195.9	-	*D*. *barbarae*	200.5
14024–0371.13	*D*. *ananassae*	196.6	-	*D*. *greeni*	201.5
14020–0011.01	*D*. *tani*	199	14028–0671.01	*D*. *jambulina*	202.7
14028–0641.00	*D*. *punjabiensis*	200.8	-	*D*. *bifasciata*	205.4
-	*D*. *phaeopleura*	202.9	-	*D*. *narragansett*	205.9
14024–0381.19	*D*. *bipectinata*	204.6	14028–0561.14	*D*. *kikkawai*	210.2
-	*D*. *malerkotliana*	204.9	14012–0161.00	*D*. *algonquin*	211
14022–0311.13	*D*. *takahashii*	207.3	-	*D*. *bicornuta*	213.7
-	*D*. *parabipectinata*	210.8	-	*D*. *diplacantha*	232.8
14028–0611.01	*D*. *orosa*	211.9	-	*D*. *biauraria*	237.2
-	*D*. *pseudotakahashii*	212.2	15085–1641.03	*D*. *hydei*	206.8
-	*D*. *mimetica*	212.7	14041–0831.00	*D*. *neocordata*	212.3
-	*D*. *serrata*	213.3	14042–0841.09	*D*. *emarginata*	214.1
-	*D*. *bunnanda*	215.2	-	*D*. *virilis*	325.4
14028–0671.02	*D*. *seguyi*	215.2	-	*D*. *nebulosa*	187.3
14028–0601.00	*D*. *nikananu*	216.1	14030–0791.01	*D*. *sucinea*	209.6
14028–0701.00	*D*. *tsacasi*	217.7	14030–0721.00	*D*. *capricorni*	211.8
-	*D*. *lutescens*	219.1	14030–0771.00	*D*. *paulistorum*	231.8
14028–0711.00	*D*. *vulcana*	222.7	-	*D*. *equinoxialis*	247.9
-	*D*. *ercepeae*	224	-	*H*. *pictiventris*	162.8
-	*D*. *pseudoananassae nigrens*	224	80000–2761.03	*S*. *leonensis*	261.8
-	*D*. *prostipennis*	227.4	-	*S*. *stonei*	206.8
-	*D*. *pseudoananassae*	228.4	11010–0021.00	*S*. *lebanonensis lebanonensis*	210.3
-	*D*. *eugracilis*	228.9	-	*S*. *pattersoni*	213.2
-	*D*. *paralutea*	230.8	50001–0001.02	*Z*. *tuberculatus*	197.6
14028–0571.00	*D*. *lacteicornis*	242.6	50000–2744.02	*Z*. *sepsoides*	212.8
14028–0471.00	*D*. *auraria*	245.1	-	-	-

Genome sizes were obtained from published literature and the laboratory database of J. Spencer Johnston. Species were obtained from the UC San Diego Stock Center. Genome size in these species were found to have a mean of 215.5 Mbp, a median of 210.8 Mbp, a minimum of 139.9 Mbp, and a maximum of 395.2 Mbp.

### Gene sequences and alignment

Sequence data for the 16 genes used to create a molecular phylogeny (4 mitochondrial and 12 protein coding genes) (*COI*, *COII*, *COIII*, *Cytb*, *Amy*, *AmyRel*, *Ddc*, *boss*, *SNF*, *Marf*, *Sod*, *per*, *Wee*, *HB*, *ADH*, and *fkh*) was downloaded from NCBI Genbank (S1 Table) and aligned using MAFFT v.7 online using iterative refinement methods (http://mafft.cbrc.jp/). Aligned sequences were visually inspected for irregularities in amino acid translation in Mesquite 2.75 and corrected by hand as needed.

### Model testing

Each sequence alignment was analyzed in JModelTest 2.1.4 to determine the model of sequence evolution that provided the best likelihood value [26]. The likelihood search assumed 11 possible substitution schemes, allowing for both invariant sites and gamma distributions. A fixed BIONJ-JC tree was used for the likelihood calculations. All runs returned the same suggested best model for phylogeny construction, a GTR substitution model with a gamma distribution and invariant sites.

### Data file preparation and tree construction

All sequences were interleaved to produce a 10,382 bp alignment. Missing sequence data was imputed for taxa that did not have gene sequences for every gene. Missing data does not influence the results of branch lengths or phylogenetic relationships. This resulted in an average of 7 genes per taxa, with a maximum of 15 and a minimum of 3 genes.

A phylogeny for *Sophophora* was reconstructed using a supermatrix model of phylogeny construction utilizing MrBayes 3.2.3 on the CIPRES supercomputer (http://www.phylo.org/) with four chains and four runs and a GTR gamma + I evolutionary model for 32,835,000 generations (sampling every 1,000) using a Dirichlet prior of (1, 0.5, 1, 1) [27, 28]. Parameter output was visualized in Tracer v 1.6 to assure the four runs had reached convergence and to determine burn-in. The consensus tree was then visualized in FigTree v.1.4.2. Genome size was mapped onto the phylogeny using the ContMap function from the phytools package from R3.3.0 [29]. Multiple trees were constructed with varying Bayesian priors to test if there were any issues with branch lengths (Dirichlet (1,1,1,1), exponential (10)) and a maximum likelihood tree.

### Tree manipulation and significance tests with different numbers of taxa

To test for the effect of taxa number on significance levels in a phylogenetic signal analyses, multiple reduced taxa phylogenies were made (5, 10, 15, 20, 30 taxa) with 20 different trees for each group. Trees were constructed by randomly trimming the taxa from the original *Drosophila* tree utilizing the drop.tip function in the package ape from R 3.3.0 [30], while maintaining tree topology and branch lengths. Taxa retained for each tree were chosen by random number generation.

### Trait analyses

Comparative phylogenetic analyses (Pagel’s parameters of evolution, Blomberg’s K, Moran’s I, and Abouheif’s C_mean_) were run on both the full phylogeny and each reduced taxa phylogeny with genome size a continuous trait. Pagel’s lambda (λ) and Blomberg’s K test for phylogenetic signal assuming Brownian motion. Pagel’s kappa (κ) tests how traits evolve along branch lengths (κ < 1, early change; κ = 1, gradual change). Pagel’s delta (δ) tests how traits change from the overall path on the tree, from root to tip (δ < 1, rapid early change, δ = 1, gradual change; δ > 1, increasing rate of change). All comparative phylogenetic analyses were completed using functions and packages available in R. Pagel’s parameters of evolution were measured using the function PGLS from package caper [31]. Blomberg’s K was estimated using the phylosignal function from package picante [32]. Moran’s I and Abouheif’s C_mean_ values were calculated using the function abouheif.moran with 999 permutations from package adephylo [33].

### Alternative test for adaptive hypothesis of genome size evolution

In order to test the alternative adaptive hypothesis for genome size, climatic data for these *Sophophora* species (critical thermal maximum, maximum temperature, minimum temperature, annual mean temperature, annual precipitation, precipitation from the wettest month, precipitation from the driest month, and latitude) were mined from two Kellerman et al. papers on phylogenetic constraint of climatic variables in *Drosophila* [34, 35]. This totaled 38 species of *Sophophora*. These variables were analyzed with multiple regression and phylogenetic generalized least squares (PGLS) analyses utilizing the function pgls from package caper [31].

### Statistical analyses

Statistical tests of fit for each comparative phylogenetic analysis is provided with output of each. For taxa number analysis, the phylogenetic values for each of the 20 runs with taxa number dataset were visualized with boxplots. The effect of taxa number on estimated phylogenetic values was tested using ANOVA in R with number of taxa treated as a random variate. In order to understand how Pagel’s λ was affected by increasing taxa number, statistical differences (p-values for H_o_: λ = 1.0 or 0.0) were plotted in a boxplot using values from each of the 20 runs at each level of reduction. These p-values were then compared with ANOVA using number of taxa as a random variate. Genome size range was also used as a covariate in an ANCOVA in R (λ p-value = Genome size range + taxa number +genome size range*taxa number) in order to see if there was an interaction between range in genome size and taxa number effect on λ significance values.

## Results

### Genome size

Genome size for the female of each species is given in Table 1. Genome size for the *Sophophora* subgenus of *Drosophila*, plus a few outgroups from *Drosophila* subgenera (*Zaprionus*, *Scaptodrosophila*, *Scaptomyza*, *Hirtodrosophila* and *Chymomyza*) ranges from 139.9 Mb to 395.2 Mb with an average of 215.5 and a median of 210.8 (Table 1).

### *Sophophora* phylogeny

**Fig 1:**
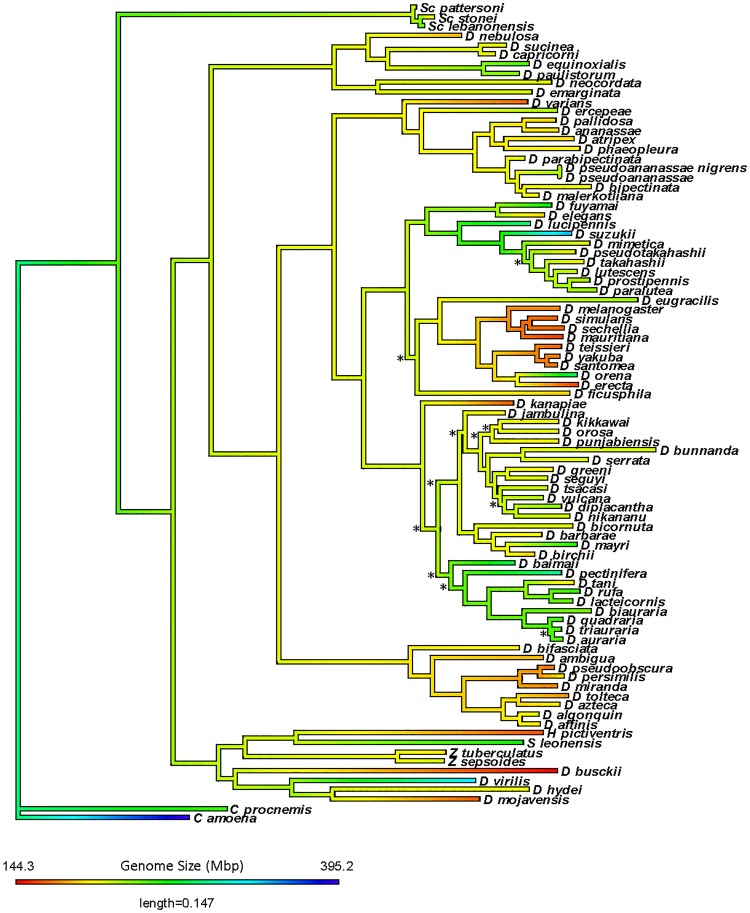
Bayesian phylogeny of *Sophophora*. Phylogeny of 87 Drosophilidae reconstructed using MrBayes 3.2.3 with a focus on *Sophophora*. Nodes with posterior probabilities lower than 80 are indicated with ‘*’. Genome size is visualized in color: smaller sizes in red, larger in blue, and intermediate in green.

The overall *Sophophora* phylogeny is well supported with high posterior probability values at each node, most being 1, with the lowest support value being 0.53 (Fig 1). The relationships in this phylogeny are supported by other large *Drosophila* phylogenies in the literature [12, 36–38]. These results suggest that the constructed phylogeny is representative of the true relationships found in *Sophophora*, and should have reliable branch lengths. No significant differences were found among trees constructed with varying Bayesian priors.

### Tests of rate and mode of genome size variation

Model fit of the complete dataset (87 genome sizes) in a phylogenetic context using the above phylogeny with branch lengths shows that phylogenetic relatedness is a significant component of genome size variation among *Sophophora*. All tests for phylogenetic signal/dependence (λ, Blomberg’s K, Abouheif’s C_mean_, and Moran’s I) indicate complete signal with high significance values (Table 2), most notably λ = 0.987. Genome size across the phylogeny was also found to have a κ value of 0.971 and a δ value of 0.589.

**Table 2:** Comparative phylogenetic output for *Sophophora* phylogeny.

Test	Value	Lower Sig.	Sig. from 1
λ	0.987	6.88E-15	0.166
δ	0.589	4.23E-10	0.174
κ	0.971	5.03E-11	1
Blomberg's K	1.373	0.001	-
Abouheif C_mean_	0.240	0.001	-
Moran's I	0.180	0.001	-

Pagel’s λ, Blomberg’s K, Abouheif’s C_mean_, and Moran’s I all found significant phylogenetic dependence for genome size in *Sophophora*. The δ value suggests an early burst of change followed by deceleration in genome size change. The κ value suggests that genome size is changing gradually on individual branches.

### Multiple regression and PGLS for climatic variables

After incorporating phylogenetic relationships, climatic variables failed to be significantly related to genome size variation in 38 species of *Sophophora*. Multiple regression analysis indicated that genome size was significantly influenced by the climatic variables (p = 0.015, Adj. R-squared = 0.30). When this model was analyzed utilizing PGLS to incorporate phylogenetic relationships, the pattern disappeared (p = 0.602, Adj. R-squared = 0.044, Table 3).

**Table 3:** Phylogenetic generalized least squares results for genome size and climatic variables.

	Estimate	Std Error	t value	Pr(>|t|)
(Intercept)	41.726	379.297	0.11	0.913
Critical Thermal Maximum	3.865	8.024	0.48	0.634
Minimum Temperature	2.303	5.032	0.46	0.651
Maximum Temperature	8.941	5.524	1.62	0.116
Annual Precipitation	-0.005	0.022	-0.25	0.807
Precipitation of Wettest Month	0.110	0.137	0.81	0.427
Precipitation of Driest Month	0.167	0.314	0.53	0.599
Latitude	-1.874	1.652	-1.13	0.266
Annual Mean Temperature	-12.545	8.622	-1.46	0.156

Residual S.E. = 166.8, Multiple R-squared = 0.1821, Adj. R-squared = -0.04358, F-statistic = 0.8068, DF = 29, p-value = 0.602

### Taxa number analyses

#### Effects on mean values

**Fig 2:**
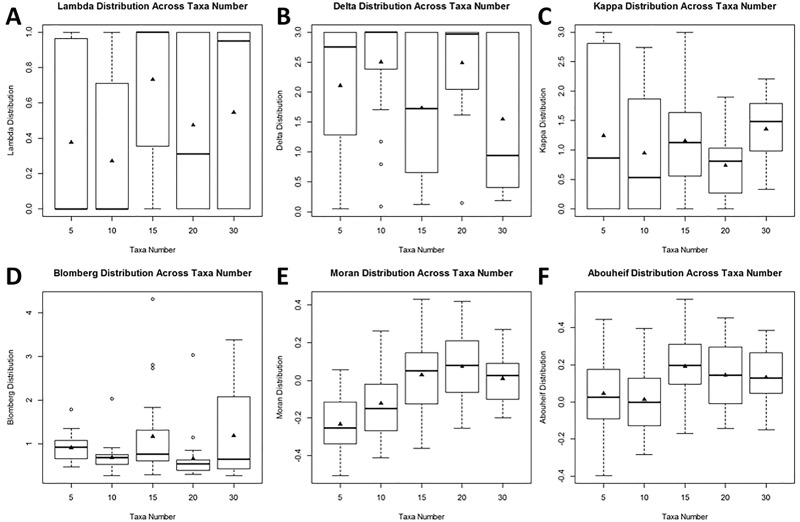
Boxplots for each phylogenetic analysis. Raw values from comparative phylogenetic tests are plotted for each group of taxa. There is no clear pattern with increasing taxa number for Pagel’s parameters of evolution or Blomberg’s K; however, there is an increase in values for both Moran’s I and Abouheif’s C_mean_. These differences are tested statistically in S2 Table.

When subsets of taxa are analyzed, means for λ, Blomberg’s K, Moran’s I, and Abouheif’s C_mean_ all increased with an increase in taxon number, indicating an increased signal of phylogenetic dependence with increased taxa number (S2 Table). However, a significant differences in the estimated parameter value for different taxon numbers was only found in Moran’s I (p = 1.67e-05) and Abouheif’s C_mean_ (p = 0.0469). No significant effect was found with increasing taxa number for λ values (Fig 2A).

#### Effects on significance

**Fig 3:**
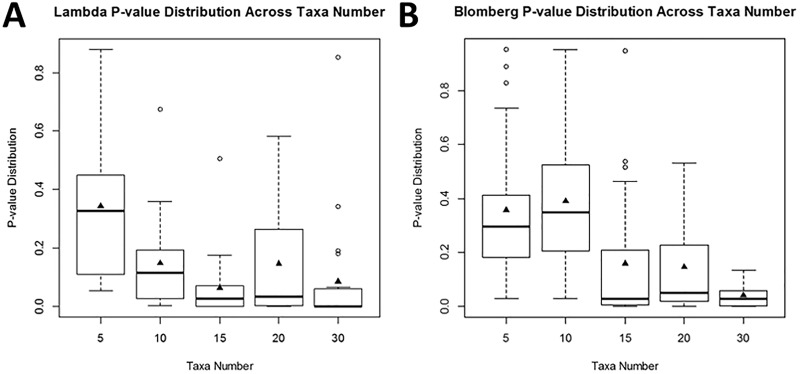
Boxplots of significance values for λ and Blomberg’s K analyses. Plotted significance values from phylogenetic signal tests of λ and Blomberg’s K decrease as the number of taxa in the analyses increase, most notably above 15 taxa. These are tested for significance in S3 Table.

In contrast to the effect of taxon number on the means, the number of significant differences of the λ estimate from the boundary conditions, 0.0 or 1.0, increased with taxa number. As shown in the boxplots, λ p-values decreased and had lower variation with increasing taxa numbers, indicating that higher taxa numbers convey higher confidence in the results for each test (Fig 3). The variation among p-values for different taxa numbers was statistically significant (p = 0.000771, ANOVA, S3 Table). A significant decrease of the p-value for Blomberg’s K was also (Fig 3) observed with increasing taxa number (p = 3.65e-07, S3 Table).

Because experimental error is expected to make up an ever larger proportion of the total variation when the measured range of genome sizes is small, we tested whether the tests of phylogenetic signal are sensitive to genome size variation among taxa. Genome size range was used as a covariate in an ANCOVA in order to determine if the range in genome size contributed to the significance of the λ results among the taxa number datasets. While the ANCOVA model was significant (p < 0.01), there was no significant interaction between genome size range and taxa number (p = 0.263). Genome size range did not contribute significantly to the model (p = 0.516), while the taxa number contribution was highly significantly (p < 0.001, Table 4).

**Table 4:** ANCOVA results for genome size range compared to λ p-values across taxa numbers.

ANCOVA for λ P-value vs. Genome Size Range and Taxa Number					
	Df	Sum Sq	Mean Sq	F value	Pr(>F)
Taxa	1	0.468	0.468	12.02	0.0008
Gsrange	1	0.017	0.017	0.42	0.5164
Taxa&Gsrange	1	0.049	0.049	1.27	0.2632
Residuals	96	3.741	0.039		

R^2^ = 0.1249, p = 0.005, f-statistics = 4.569. An ANCOVA indicates that there is no interaction between taxa number and genome size range. Only taxa number contributed significantly to the model.

## Discussion

Here, we use a comparative phylogenetic approach to investigate genome size in *Sophophora* species. We specifically look at measures of phylogenetic signal (Pagel’s λ, Blomberg’s K, Abouheif’s C_mean_, and Moran’s I) and at measures of mode and tempo of evolution (Pagel’s δ and κ) in order to test three hypotheses of genome size evolution: low effective population size hypothesis, mutational equilibrium hypothesis, and an adaptive hypothesis.

Genome size was found to have complete phylogenetic signal for *Sophophora* (λ = 0.987, Blomberg’s K = 1.373, Abouheif’s C_mean_ = 0.240, Moran’s I = 0.180, Table 2). Based on our expectations for the adaptive hypothesis, the presence of complete phylogenetic signal suggests that little, if any, of the genome size variation is evolving in an adaptive fashion. This conclusion is also supported by the results of the PGLS analysis, which found that genome size is not significantly related to climatic variables (Table 3). Interestingly, when these climatic variables were phylogenetically analyzed by Kellerman et al., they were found to have complete phylogenetic signal [34, 35]. Since, genome size and climatic variables both have phylogenetic signal, we can assume that any patterns we see between these characteristics in *Drosophila* in a non-phylogenetic aspects are due to constraints of the phylogeny, not a direct relationship. Also, when inspecting the trait mapped on the phylogeny, there is not significant evidence for bursts of change in concordance with the adaptive hypothesis, aside from the decrease in the *D*. *melanogaster* clade (Fig 1). Rather, genome size evolution is reliant upon phylogenetic patterns. These results are supported by recent work on genome size evolution in *Drosophila* species [39]. Here, Sessegolo *et al*. investigated the impact of phylogeny on genome size and transposable elements for 26 species of *Drosophila* utilizing available sequences and a de novo transposable element assembly approach. They found a significant correlation between genome size and global transposable element content, with strong phylogenetic signal for each. While simple repeats accounted for up to 1% of the repeatome, LTRs and LINE elements were found to be major components. These data suggest that the genome size variation of *Drosophila* species are largely driven by transposable elements. The current study, while not including information on proportions or dynamics of repeat sequences, has largely expanded the number of taxa used an earlier study from 26 to 87. By increasing the number of taxa, we can hope to determine if the overarching patterns of genome size evolution in *Drosophila* remain consistent and better identify which species may be of interest for full sequence investigation.

Genome size was also found to have a κ value of 0.971 (Table 2), indicating that genome size evolves in a gradual fashion and reflects individual branch lengths. The phylogenetic signal, the gradual manner in which genome size is changing, and the relationship of branch length and amount of change supports the mutational equilibrium hypothesis [8]. However, there have been concerns expressed about this hypothesis, as it seems to be largely theoretical and has yet to have a large enough dataset to support it [15, 16]. Small imbalances between insertions and deletions are not likely to move fast enough to change genome size dramatically, especially when it seems as if genome size is being driven by relatively large insertions of transposable elements [39]. The recent accordion model shows that dynamic changes in transposons may be associated with large deletions and lead to apparent stasis of genome size [17]. However, transposition and large deletions, if imbalanced, would drive genome size evolution at an accelerated rate. However, neither stasis nor an imbalance of transposition and large deletions would necessarily produce the phylogenetic signal observed for these 87 species.

Interestingly, a δ value of 0.589 (Table 2) indicates that the rate of genome size change was not always constant. This δ value suggests change occurred rapidly early in the phylogeny, likely at the formation of the *Drosophila* genus, with a decrease in that rate as time went on throughout *Sophophora*. The early change in genome size could be due to low population sizes, which would appear to support the effective population size hypothesis [6]. However, genome size in this group has moved towards smaller sizes rather than increased sizes [14], contradicting the hypothesis that lower effective population sizes lead to larger genome sizes. The original burst could therefore be adaptive. If so, the smaller genome sizes could have been due to selection on one of their phenotypic correlates [40]. Specifically, selection could have acted on smaller cell and body sizes or shorter development and cell cycle times [41–45]. It is also possible that the low effective population was inefficient at selecting out a slightly deleterious, non-adaptive trait, such as increased deletion rate. After early, relatively large genome size changes, the rate of evolution could have slowed to the current, gradual rate. It is important to note that the change in rate would have had to happen quickly to not be reflected in the κ values.

It is important to ask if further sampling will change the conclusions above. If the change in *Sophophora* genome size does actually fit the mutational equilibrium hypothesis, it is possible that heavier sampling of the genus or subgenus could fill in the gaps for the large change early in the tree. While this is a possibility, taxon sampling issues addressed in this study, suggest that the significance values and the magnitude of these values vary little when overall study size reaches n = 30. The number of taxa examined here (n = 87) is well above that. The importance of a large enough sample size for tests of phylogenetic signal cannot be ignored. Increases in significant measures of phylogenetic signal with taxa number were found to increase with taxon number in both Abouheif’s C_mean_ and Moran’s I. This suggests the results of these two methods are sensitive to taxa number and they should be used sparingly, more so as preliminary tests for comparative studies. At the same time, while Abouheif’s C_mean_ and Moran’s I were sensitive to increasing taxa number, there were no significant effects of taxa number on either Pagel’s λ or Blomberg’s K, suggesting the results of these methods are less sensitive to taxa number. At the same time, while there are no clear patterns for the magnitude of the parameter values of phylogenetic signal, there is a significant difference in the p-values obtained at different taxa numbers. Since the p-values are measures of significant differences from the bounds (signal vs. no signal), they can be considered proxies for test reliability. Based on these analyses, sample sizes of at least 15 are necessary to achieve reliable results in terms of significance for Pagel’s λ and Blomberg’s K (Fig 3). The pattern of increased reliability (statistical p-value from the bounds) continues as the taxa number increases; the best results are obtained with larger taxon sampling. These results are supported by a previous study tested the effectiveness of detecting phylogenetic signal using simulated taxa with ranges of Brownian motion [46].

The number of taxa is important, yet the range in the trait value across the tree could also affect the reliability of the phylogenetic signal results. Narrow or wide ranges in variation could skew the interpretation of these comparative results. However, we found that sampling from the range of genome size in *Sophophora*, had a non-significant effect (p = 0.5164) and no significant interaction was found between genome size range and taxa numbers (p = 0.263). Only taxa number was found to be significantly contributing to the fit of the ANCOVA model (Table 4). Reduced taxa results, in conjunction with previous results using simulated datasets [46], show the strength of these tested methods for calculating phylogenetic signal. Most emphasis should be put into Blomberg’s K and Pagel’s parameters of evolution, as they are least sensitive to taxa number affecting the calculated phylogenetic signal value. However, these two methods must have at least a minimal sample size (15–20) to achieve reliable results. While there are some taxa number effects on phylogenetic signal estimates for Abouheif’s C_mean_ and Moran’s I, they still are good quick, preliminary measures for phylogenetic signal before the use of more robust comparative methods, such as Pagel’s λ and Blomberg’s K.

While the signal detected here rejects a non-phylogenetic model of change, it has yet to fully support one of the proposed phylogenetic patterns of change (effective population size vs. mutational equilibrium). The early burst of change (δ = 0.589) would seem to fit the small species effective size hypothesis, yet the trend is to a decrease rather than an increase in genome size, suggesting that this change could be due to adaptation or selection. The gradual change (κ = 0.971) in genome size after that burst suggests a model similar to the mutational equilibrium hypothesis with large deletions balancing out the large insertions due to transposable elements. We argue therefore, that the rapid early change in *Sophophora* may represent an increase in deletion rate, and possibly an adaptive radiation associated with selection for rapid development rate and small size. Subsequent change is gradual as expected of a deletion insertion balance.

## Supporting information

S1 Table Table of accession numbers for phylogeny. (XLSX)

S2 Table ANOVA results and means for each phylogenetic analysis. Phylogenetic value means from each phylogenetic analyses for taxa number (5, 10, 15, 20, and 30 taxa) datasets were compared using ANOVA. Given the results, Moran’s I and Abouheif’s Cmean are significantly affected by taxa number.
(XLSX)

S3 Table ANOVA and means for P-values for λ and Blomberg’s K analyses. P-values from the opposite bounds for Pagel’s λ and Blomberg’s K for the taxa number (5, 10, 15, 20, and 30 taxa) datasets were compared using ANOVA. Given the results, the reliability of these phylogenetic signal tests increase as taxa number increases.
(XLSX)
